# Improving protein function prediction using domain and protein complexes in PPI networks

**DOI:** 10.1186/1752-0509-8-35

**Published:** 2014-03-24

**Authors:** Wei Peng, Jianxin Wang, Juan Cai, Lu Chen, Min Li, Fang-Xiang Wu

**Affiliations:** 1 School of Information Science and Engineering, Central South University, Changsha, Hunan 410083, PR China; 2 Faculty of Information Engineering and Automation, Kunming University of Science and Technology, Kunming, Yunnan 650093, PR China; 3 Department of Mechanical Engineering and Division of Biomedical Engineering, University of Saskatchewan, Saskatoon, SK S7N 5A9, Canada

## Abstract

**Background:**

Characterization of unknown proteins through computational approaches is one of the most challenging problems in silico biology, which has attracted world-wide interests and great efforts. There have been some computational methods proposed to address this problem, which are either based on homology mapping or in the context of protein interaction networks.

**Results:**

In this paper, two algorithms are proposed by integrating the protein-protein interaction (PPI) network, proteins’ domain information and protein complexes. The one is domain combination similarity (DCS), which combines the domain compositions of both proteins and their neighbors. The other is domain combination similarity in context of protein complexes (DSCP), which extends the protein functional similarity definition of DCS by combining the domain compositions of both proteins and the complexes including them. The new algorithms are tested on networks of the model species of *Saccharomyces cerevisiae* to predict functions of unknown proteins using cross validations. Comparing with other several existing algorithms, the results have demonstrated the effectiveness of our proposed methods in protein function prediction. Furthermore, the algorithm DSCP using experimental determined complex data is robust when a large percentage of the proteins in the network is unknown, and it outperforms DCS and other several existing algorithms.

**Conclusions:**

The accuracy of predicting protein function can be improved by integrating the protein-protein interaction (PPI) network, proteins’ domain information and protein complexes.

## Background

The function annotation of a protein is an important challenge in post-genomics due to the critical roles of proteins in various biological processes. However, it is expensive and time-consuming to experimentally determine protein functions. With rapid advances in large scare genome sequencing technologies, there is an increasingly widening gap between the number of newly found proteins and the completeness of their annotations, which requires a faster and more effective way to annotate unknown proteins automatically. Hence, the protein function prediction through computational approaches has become a major research topic, which has drawn much attention from researchers in the areas.

Computationally predicting protein function is based on the idea that assigning functions to unknown proteins according to the known functions of similar proteins. The most common and reliable methods are using homology mapping to transfer annotations to newly sequenced proteins. One of the way to infer to homology is detecting sequence similarity by using BLAST [1] and FAST [2]. Another is to identify protein domains by using the databases or tools, such as Pfam [3], PRODOM [4], SCOP [5] and so on. Domains are some compactly structured components of a protein that can evolve, function, and exist independently of the rest of the protein chain. The vast varieties of protein functions can be derived from the different combinations and cooperation of protein domains [6]. Therefore, there are methods [7,8] where the proteins’ internal domain compositions are compared directly without considering the whole sequence. The homology mapping approach is based on the assumption that homologous proteins have most likely evolved from a common ancestor and thus must have similar functions. However the weakness of this kind of methods is that few un-annotated proteins hit to known proteins as the data of sequenced proteins continue to expand at the exponential rate.

On the other hand, with the increase of large scale protein-protein interaction (PPI) data generated by two-hybrid and co-immunoprecipitation techniques, many researchers have attempted to determine protein functions by using information extracted from the PPI data [9]. Existing computational methods based on PPI can be roughly divided into two main categories: direct methods that straightforwardly utilize the protein interactions and module-assisted schemes that use function modules to infer protein functions as a whole [9].

Direct methods are based on the fact that about 70% to 80% of proteins share at least one common function with their interacting partners [10]. One of the earliest these methods is neighborhood counting method proposed by Schwikowski et al. [10]. The method counts up the times of a function occurring in the protein’s neighborhood to estimate the possibility that a given function can be assigned to an un-annotated protein. However, this method ignores the background frequency of different function annotations. In reference [11], the authors have tried to improve the original neighbor counting method by computing the Chi-square statistics as an indicator of the statistical significance of the function under consideration. Vazquez et al. have assigned functions to proteins via a global mechanism that maximizes the number of edges that connect proteins with the same function in their paper [12]. Recently, an iterative method has been introduced to make the prediction of functions iteratively to get a most consistent agreement throughout the whole network [13]. There are also some other algorithms for global function assignments described in [14-16]. Considering that previous methods predict the function of an un-annotated protein only relying on direct neighbors, Chua et al. have investigated the functional information within both direct and indirect neighbors by giving them different weights [17]. Based on the observation in ref. [17], this group propose a topological weight, FS-Weight, which estimates function association between direct and indirect interactions, to infer protein functions [18] and predict protein complexes [19]. Due to high noise-signal ratio of protein-protein interaction data, those direct methods which infer protein functions in terms of protein interactions may not work well. To overcome this problem, some researchers [7,16,20-22] either have combined multiple network information resources, such as expression profiles, gene regulatory networks, PPI networks, GO similarity network and so on, or have used a wide variety of biological characteristics, including sequence patterns, homology data, previously known functional annotation, protein complex and so on. Lin et al. [23] have proposed a novel common-neighbor based model and a Bayesian framework to predict protein functions. Their studies have shown that two proteins are likely to have same functions if they share common neighbors, and the more common neighbors they have, the more likely they have same functions. Zhang et al. [24] have extended the concepts of common neighbors to the domain compositions of proteins’ neighbors, which they introduce as domain contexts. They believe that similar domain compositions inside the neighbors may indicate both functional similarity and evolutionary relationship, and define a domain context similarity to assess the function similarities between proteins. For simplicity, we name their method as Zhang-DC. However, this method has not considered the similarity of proteins themselves.

Instead of predicting functions for each protein separately, module-assisted methods first identify the function modules or protein complexes, and then annotate functions to all proteins in the function modules or protein complexes. Although the module-assisted methods vary in the clustering algorithms for identifying the function modules or protein complexes, they are based on the fact that functional module means a group of cellular components and their interactions that can be attributed to a specific biological function [25]. Previous studies [26] have pointed out that module-assisted methods are most useful in networks obtained from genomes with few protein annotations. It suggests that we can infer protein functions by using the whole function modules or protein complexes while not only limited to the direct interactions.

With respect to the above issues, we propose a new algorithm by defining a domain combination similarity in PPI networks as a measurement of the protein function similarity, named by DCS. In DCS, the protein functional similarity combines the domain compositions of both proteins and their neighbors. Then we propose another new algorithm DSCP which extends the protein functional similarity definition in DCS by combining the domain compositions of both proteins and complexes including them. Differently from previous homology mapping approach, our methods integrate the PPI data information and protein complex information. Differently from previous methods based on PPI data, our methods combine the domain information of protein itself and take protein complex information for consideration. We carry out experiments on data from *Saccharomyces cerevisiae*. As the comparisons are shown, our methods can make an apparent improvement on the performances of function prediction than other methods, such as neighbor counting [10], Chi-square [11], Zhang-DC [24], Markov random field (MRF) model [7] and relaxation labelling classifier (RLC) [16].

This paper is organized as follows: First of all, recent advances of function prediction algorithms are introduced briefly. Then the relevant definitions as well as the new method are described in details. In the third section, the materials used in this paper are given and we present the results produced by our new algorithms and a comprehensive comparison between the new algorithms and five other existing algorithms. Finally, challenges and directions of the future work are discussed in Conclusions and discussion.

## Methods

When we predict the functions for an unknown protein, firstly the function similarities between proteins are defined. Then we find out a known protein with the highest functional similarity value to the unknown protein from the network, and the functional annotations of the known protein are then assigned to the unknown protein. In this work, we propose two algorithms to evaluate the functional similarity between two proteins. The one is DCS, the domain combination similarity, which combines the domain compositions of both proteins and their neighbors. The other is DSCP, which extend the protein functional similarity definition of DCS by combining the domain compositions of both proteins and complexes including them.

### Domain combination similarity (DCS)

**Figure 1:**
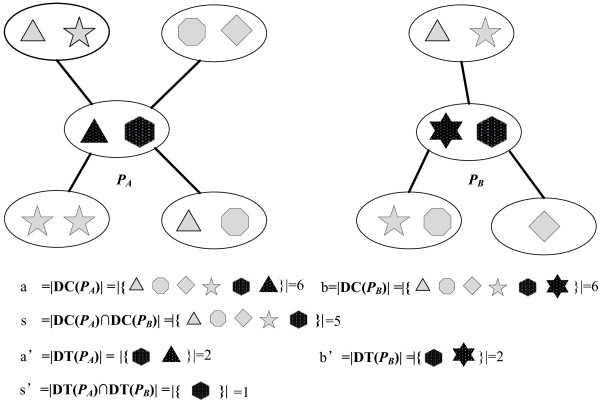
***Illustration of domain combination similarity.*** The figure gives an example of the domain combination similarity of protein *P*_*A*_ and *P*_*B*,_ in which different shapes are drawn to represent different types of domains. The domain combination similarity of the two proteins is consisted of two parts: the context similarity, which is presented in the figure as the light grey part plus the dark black part, and the composition similarity, which is presented in the figure as the dark black. Domain composition of protein *P*_*A*_*,* denoted by DT(*P*_*A*_), is a set of domain types in protein *P*_*A*_. Domain context of *P*_*A*_, denoted by DC(*P*_*A*_), is a set of distinct domain types in the neighbor proteins of *P*_*A*_ (*P*_*A*_ included).

Our measurement of protein domain combination similarity is consisted of two parts, the context similarity, which indicates the domain similarity of proteins’ neighbors, and the composition similarity, which indicates the proteins’ internal domain similarity. The context similarity is presented in Figure 1 as the light gray part plus the dark black part, while the composition similarity corresponds to the dark black part in Figure 1. These two parts will be added up to get a final estimation of the function similarity between two proteins. As can be seen in Figure 1, it is the same five kinds of domain types that are contained by the neighbors of protein *P*_*A*_ and *P*_*B*_ in spite of their different distributions while it is the same one kind of domain type that is contained by protein *P*_*A*_ and *P*_*B*_, which indicates *P*_*A*_ and *P*_*B*_ probably share similar functions. To formally define the domain combination similarity, the following variables are introduced.

Given a PPI network, let *SN* = {*P*_1_, *P*_2_, …, *P*_*n*_} represent a set of all *n* proteins in the PPI network and *N*_*P*_ denotes a set of neighbor proteins of protein *P* with *P* itself included.

Let DT(*P*) denote a set of domain types in protein *P*. Given a protein set *S* = {*P*_*s*1_, *P*_*s*2_, …, *P*_*sl*_}, we define

(1) $$\mathrm{DT}\left(S\right)=\cup \mathrm{DT}\left(P_{i}\right),\;i=s_{1},\ldots ,s_{l}.$$

Let DC(*P*) be a set of distinct domain types in the neighbor proteins of *P* , which is called the domain context of *P*. Consequently we have

(2) $$\mathrm{DC}\left(P\right)=\mathrm{DT}\left(N_{P}\right).$$

Let *M* denote the number of domain types in the whole network, and let *a* and *b* represent the number of domain types in the neighbors of *P*_*A*_ and *P*_*B*_, respectively. Let *s* denote the number of common domain types in their neighbors. Then we can get the following equations:

(3) $$\begin{array}{l} M=|\mathrm{DT}\left(\mathrm{SN}\right)|,\\ a=|\mathrm{DC}\left(P_{A}\right))|,b=|\mathrm{DC}(P_{B})|,\\ s=|\mathrm{DC}\left(P_{A}\right)\cap \mathrm{DC}(P_{B}))|. \end{array}$$

The domain context similarity, *f*_*cont*_ , can be defined as follows.

(4) $$f_{\mathrm{cont}}\left(P_{A},P_{B}\right)=-\log\frac{\left(\begin{array}{c} M\\ s \end{array}\right)\left(\begin{array}{c} M-s\\ a-s \end{array}\right)\left(\begin{array}{c} M-a\\ b-s \end{array}\right)}{\left(\begin{array}{c} M\\ a \end{array}\right)\left(\begin{array}{c} M\\ b \end{array}\right)}$$

where $\left(\begin{array}{c} m\\ n \end{array}\right)$ denotes combinatorial numbers.

Let *a’* and *b’* denote the number of domain types inside protein *P*_*A*_ and protein *P*_*B*_ respectively, and let *s’* denote the number of common domain types of *P*_*A*_ and *P*_*B*_. Then we can get the following equations:

(5) $$\begin{array}{l} a'=|\mathrm{DT}\left(P_{A}\right)|b'=|\mathrm{DT}(P_{B})|\\ s'=|\mathrm{DT}\left(P_{A}\right)\cap \mathrm{DT}(P_{B})|. \end{array}$$

Similarly to *f*_*cont*_, the domain composition similarity, *f*_*comp*_, can be defined as follows:

(6) $$f_{\mathrm{comp}}\left(P_{A},P_{B}\right)=-\log\frac{\left(\begin{array}{c} M\\ s' \end{array}\right)\left(\begin{array}{c} M-s'\\ a'-s' \end{array}\right)\left(\begin{array}{c} M-a'\\ b'-s' \end{array}\right)}{\left(\begin{array}{c} M\\ a' \end{array}\right)\left(\begin{array}{c} M\\ b' \end{array}\right)}$$

We believe that these two parts should not be treated equally. Alternatively, they are added together via a parameter *λ*, and finally, the domain combination similarity between two proteins *P*_*A*_ and *P*_*B*_ is defined as below:

(7) $$f_{\mathrm{sim}}\left(P_{A},P_{B}\right)=\lambda *f_{\mathrm{cont}}\left(P_{A},P_{B}\right)+\left(1-\lambda \right)*f_{\mathrm{comp}}\left(P_{A},P_{B}\right).$$

A larger value of *f*_*sim*_(*P*_*A*_, *P*_*B*_) between two proteins *P*_*A*_ and *P*_*B*_ will indicate a greater probability that they share similar functions.

For the convenience of discussion, in the following sections DCS (Domain Combination Similarity) will be adopted to calculate the current definition of *f*_*sim*_ for protein function prediction. The pseudo code for calculating the functional similarity *f*_*sim*_(*P*_*A*_, *P*_*B*_) between two proteins *P*_*A*_ and *P*_*B*_ using algorithm DCS is presented below.

### Domain combination similarity in context of protein complexes (DSCP)

We argue that the original manner of taking neighbors as the domain context in DCS can be further improved by using the protein complexes information instead. Since the PPI network is not complete and has false interactions due to experimental limits and errors, merely considering the neighborhood can produce bias results. For the sake of common noises in the interaction data, when calculating the domain context similarity *f*_*cont*_, we don’t just consider the neighbors of a protein as in most classical algorithms but also search for the complexes containing the proteins. Here it is believed that functionally similar proteins tend to cluster together and protein complexes are this kind of collections of functionally related proteins [27-31]. There are many algorithms that try to make use of protein complex data to infer protein functions [7,32]. Deng et al. [7] have used an MRF model to integrate multiple sources of data including the protein complexes while Joshi et al. [32] have made use of protein complexes by assigned binary interactions to two proteins involved in a same protein complex and developed an integrated probabilistic method for cellular function prediction. Consequently, by means of integrating protein complexes to serve as the domain context scope, it promises to get a better measurement of protein function similarity.

Let *SC*_*P*_ be the set of all proteins inside those complexes containing protein *P*. Then the previous definition of domain context of *P* is adjusted as follows:

(8) $$\mathrm{DC}\left(P\right)=\mathrm{DT}\left(SC_{P}\right).$$

The formulas defining *f*_*cont*_, *f*_*comp*_ and *f*_*sim*_ still remain the same as before. Again a larger value of *f*_*sim*_ indicates a greater probability of sharing similar functions.

With regard to the protein complex data, we can use either known real protein complexes from assays or protein complexes predicted by various network clustering algorithms. Since both the known protein complexes and predicted protein complexes have the limited coverage for the original proteins in the whole network, we will still use the neighborhood as the context scope, if a protein is not included in any complex. We call the prediction algorithm based on this new definition as DSCP (Domain combination Similarity in context of protein complexes). The following pseudo code illustrates the procedure of calculating the functional similarity by using algorithm DSCP.

### Protein function prediction

Given the protein similarity definitions described above, the functional similarities *f*_*sim*_ between each pair of proteins can be calculated conveniently. When predict the functions for an unknown protein *P*_*u*_, we find out a known protein *P*_*m*_ with the highest value of *f*_*sim*_ to *P*_*u*_ from the network, and the function annotations of *P*_*m*_ are then assigned to *P*_*u*_. If there exist more than one protein that are of the same highest *f*_*sim*_ to *P*_*u*_, the first coming one will be selected as the reference.

To evaluate the performances of a predicting algorithm, the cross validation is generally used [24,33,34]. All the proteins in the PPI network are partitioned into two subsets, the training set and the testing set. In one round of cross validation, the functions of each protein in the testing set are predicted according to the proteins in the training set. The validation process is performed multiple times to make sure that each sample will have a chance as a member of the testing set once. The final performances are averaged over all rounds. There are several partition schemes. Some studies [24] use leave-one-out cross validation which put one protein into the testing set and the remaining proteins into the training set, while other studies [33,34] use leave-percent-out cross validation, which randomly selects a percentage of proteins as the testing set and then puts other proteins into the training set.

In a binary classifier system, there are four types of possible outcomes for each prediction, namely, true positive (*TP*), true negative (*TN*), false positive (*FP*) and false negative (*FN*). *TP* and *TN* are the correct predictions while *FP* and *FN* are the two kinds of wrong classifications. *FP* is a positive prediction that is in fact negative and *FN* is a negative prediction that is actually positive. Therefore, in some studies [24], there are three measurements are generally used to assess the qualities of prediction algorithms: precision (also called positive predictive value and denoted as *PPV*), recall (also called sensitivity or true positive rate and denoted as *TPR*), *F*-Measure and Matthew correlation coefficient (denoted as *MCC* and ranging from -1 to 1 with a larger *MCC* value indicating a better prediction results). The three measurements are defined as follows.

(9) $$\mathrm{PPV}=\frac{\mathrm{TP}}{\mathrm{TP}+\mathrm{FP}},$$

(10) $$\mathrm{TPR}=\frac{\mathrm{TP}}{\mathrm{TP}+\mathrm{FN}},$$

(11) $$F-\mathrm{Measure}=\frac{2*\mathrm{PPV}*\mathrm{TPR}}{\mathrm{PPV}+\mathrm{TPR}},$$

(12) $$\mathrm{MCC}=\frac{\left(\mathrm{TP}\times \mathrm{TN}\right)-\left(\mathrm{FN}\times \mathrm{FP}\right)}{\sqrt{\left(\mathrm{TP}+\mathrm{FN}\right)*\left(\mathrm{TN}+\mathrm{FP}\right)*\left(\mathrm{TP}+\mathrm{FP}\right)*\left(\mathrm{TN}+\mathrm{FN}\right)}}$$

The process of function prediction using leave-one-out cross validation is described below.

## Results

The *Saccharomyces cerevisiae* (yeast) protein interaction networks are widely used as a gold standard data in the research of network-based function prediction algorithms because the species of yeast has been studied most widely and thus the available interaction data for yeast is the most complete and convincible. Here, we also adopt yeast interaction network to test our new algorithms. The PPI network data is obtained from DIP database [35]. The annotation data of proteins used for algorithm validation is the latest version (*2012.3.3*) downloaded from GO official website [36] as is the same case with the domain data from Pfam database [3] (*26.0*). As for the protein complex information, we used the data of CYC2008 [37] which consists of 408 protein complexes involving 1,439 proteins in yeast obtained by reliable manual curation.

The original interaction networks are transformed to use the UniProtKB/Swiss-Prot entries because the Pfam domain data use such labelling system. Consequently a network of 5,088 proteins and 22,277 interactions is obtained after removing the self-interaction and some proteins without UniProtKB/Swiss-Prot entries.

The prediction performance is validated based on Gene ontology (GO) annotations. The GO system consists of three separate categories of annotations, namely Molecular Function (MF), Cellular Component (CC) and Biological Process (BP). The Predictions are validated separately for each of the three GO categories. Since the GO terms are organized as a hierarchical structure, in which a protein that is annotated with a GO term is also annotated with all its ancestors, using all GO terms for validation may result in biased conclusions. To avoid too special and too general [24], only those GO terms that annotate at least 10 and at most 200 proteins will be kept in the experiments. Moreover we adopt the same way of selecting reliable GO terms as previous study [38] and ignore the GO terms that are annotated with evidence code IEA (Inferred from Electronic Annotation), ND (No biological Data available), NAS(Non-traceable Author Statement). Therefore, the final gold standard consists 95, 124, 267 GO terms for MF, CC and BP respectively.

Moreover, we only use the Pfam-A data because the Pfam-A data have been manually checked and thus more convincible while Pfam-B part is automatically generated by HMM computational methods [3]. In the finally data sets, among the 5,088 proteins, there are 4,260 proteins with domain information and 2895, 3868 and 3909 proteins annotated by at least one GO term in MF, CC and BP respectively.

### Parameters determination and data analysis

**Figure 2:**
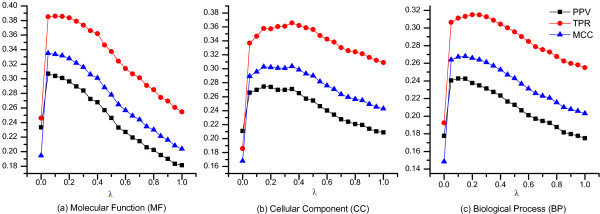
***The distribution of average PPV, TPR and MCC on different values of λ in algorithm DCS.*** The figure depicts the distribution of average *PPV*, *TPR* and *MCC* when different values of λ are selected in algorithm DCS. X-axis represents the different values of λ. Y-axis represents the values of each performance measure (*PPV*, *TPR* and *MCC*). **(a)**, **(b)** and **(c)** illustrate the results based on GO terms in MF, CC and BP respectively.

In our definition of domain combination similarity, a parameter *λ* is introduced to adjust the two parts of *f*_*cont*_ and *f*_*comp*_. With different values of parameter *λ*, the performances of prediction might differ greatly. As a result, we investigate the effect of parameter *λ* on algorithm DCS by running 20 times with equal interval of *λ* from 0 to 1. The corresponding indices of *PPV*, *TPR* and *MCC* at different value of *λ* are calculated. The results based on GO terms in MF, CC and BP are illustrated in Figure 2 (a), (b) and (c) respectively. Figure 2 shows that the performance of DCS when *λ* is either 0 or 1 is inferior to when *λ* is set to other values ranging from 0 to 1, which means that the prediction performance by integrating the context similarity and the composition similarity is better than that by using only either similarity. For each one of the three GO annotation categories, in terms of values of *PPV*, *TPR* and *MCC*, the performance of DCS rises sharply when *λ* is 0.5 and drops rapidly when *λ* is larger than 0.4. Although the values of *λ* vary a litter when the values of *PPV*, *TPR* and *MCC* reach their peak, DCS remains high performances when *λ* is in the range of 0.05 to 0.35. Therefore, we use *λ* = 0.1 as a default value in all the following experiments.

**Figure 3:**
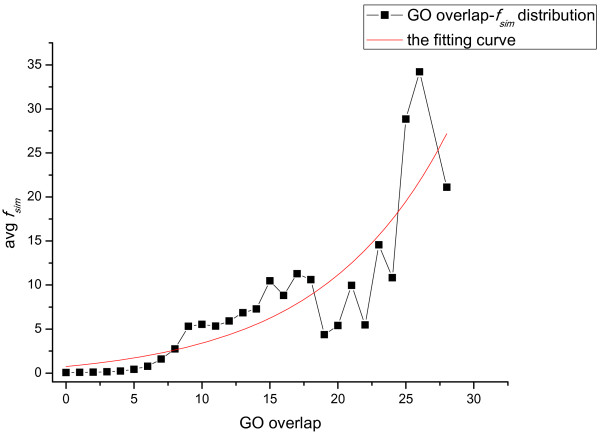
**The distribution of domain combination similarities with regard to different function similarities.** The figure illustrates the statistical results of the average domain combination similarities with regard to different function similarity values. The function similarity value of two proteins is presented in terms of the overlaps of the GO terms between them. X-axis represents function similarities. Y-axis represents the corresponding the average domain combination similarities (avg *f*_*sim*_) between pairs of proteins in the network.

Then, we make a statistic on the average domain combination similarities with regard to different function similarity values which are presented in terms of the overlaps of the GO terms between proteins. Firstly, the domain combination similarities between each pair of proteins in the network are calculated using the definition of DCS with *λ* set as 0.1. Then they are averaged over different values of GO overlaps to get a distribution curve. As can be seen in Figure 3, when the overlap of GO terms increases, which means the function similarity increases, the domain combination similarities between proteins tend to increase as well. Consequently, we conclude that our defined domain combination similarity can be a good indicator for the functional relations between different proteins.

### The results of function prediction

We test the qualities of our algorithms for predicting protein functions using the leave-one-out cross validation, which means for each round there is only one protein in the testing set. Note that we will filter out those GO terms whose sizes are smaller than 10 or greater than 200 because they are either too rare or too general for prediction. Here, the size of GO term refers to the number of proteins in each GO term. Moreover, experiments will be implemented separately for MC, CC and BP. Therefore, GO terms in each annotation category have been partitioned into four groups based on their positive size. The top part of Table 1 shows the results of function prediction on DIP PPI network when using the algorithm DCS.

**Table 1:** The results of DCS, DSCP and DSCP using protein complexes predicted by DPClus on DIP PPI

**Methods**	**Size**	**MF**					**CC**					**BP**				
		**#GO**	**PPV**	**TPR**	**F-measure**	**MCC**	**#GO**	**PPV**	**TPR**	**F-measure**	**MCC**	**#GO**	**PPV**	**TPR**	**F-measure**	**MCC**
DCS	[10–30]	75	0.44	0.37	0.40	0.40	89	0.40	0.39	0.39	0.38	216	0.34	0.35	0.34	0.35
	(30–50]	10	0.41	0.36	0.39	0.38	18	0.30	0.25	0.27	0.26	36	0.31	0.30	0.30	0.30
	(50–100]	8	0.50	0.42	0.46	0.45	12	0.33	0.30	0.32	0.30	13	0.34	0.33	0.33	0.33
	(100–200]	2	0.71	0.62	0.66	0.65	5	0.27	0.24	0.25	0.22	2	0.47	0.46	0.47	0.46
	In total	95	0.45	0.38	0.41	0.41	124	0.37	0.35	0.36	0.35	267	0.34	0.34	0.34	0.34
DSCP	[10–30]	75	0.44	0.36	0.40	0.39	89	0.49	0.48	0.48	0.48	216	0.38	0.38	0.38	0.37
	(30–50]	10	0.37	0.31	0.34	0.33	18	0.36	0.33	0.34	0.33	36	0.37	0.35	0.36	0.35
	(50–100]	8	0.50	0.38	0.43	0.42	12	0.35	0.31	0.32	0.31	13	0.40	0.40	0.40	0.39
	(100–200]	2	0.73	0.55	0.63	0.62	5	0.30	0.26	0.28	0.25	2	0.55	0.53	0.54	0.53
	In total	95	0.44	0.36	0.40	0.39	124	0.45	0.43	0.44	0.43	267	0.38	0.37	0.38	0.37
DSCP_DPClus	[10–30]	75	0.44	0.36	0.40	0.39	89	0.41	0.34	0.37	0.36	216	0.34	0.32	0.33	0.33
	(30–50]	10	0.37	0.31	0.34	0.33	18	0.30	0.25	0.27	0.26	36	0.30	0.26	0.28	0.27
	(50–100]	8	0.50	0.38	0.43	0.42	12	0.32	0.30	0.31	0.29	13	0.35	0.30	0.33	0.31
	(100–200]	2	0.73	0.55	0.63	0.62	5	0.26	0.25	0.26	0.23	2	0.49	0.48	0.48	0.47
	In total	95	0.44	0.36	0.40	0.39	124	0.38	0.32	0.34	0.34	267	0.34	0.32	0.33	0.32

The GO terms in MF, CC and BP are divided into 4 groups according to the number of proteins annotated by them, respectively. Those GO terms which are consisted of more than 10 or less than 200 proteins are filtered out because they are either too rare or too general for prediction. This table shows prediction results of DCS, DSCP and DSCP using protein complexes predicted by DPClus (DSCP_DPClus) based on DIP PPI data, including the number of GO terms in each group and each category, and the corresponding values of *PPV*, *TPR* and *MCC*. Here, column "size" means the number of protein in each GO term. "#GO" denotes the number of GO terms.

Next, the algorithm of DSCP is adopted to predict protein function on the same PPI data sources by using the experimentally determined protein complexes obtained from CYC2008. 966 of 2895, 1281 of 3868 and 1314 of 3909 proteins that are annotated by a least one GO term in MF, CC and BP respectively present in at least one CYC2008 complex. For those proteins belonging to CYC2008 complex, DSCP infers their domain context by searching for the complexes containing them, whereas the domain context of the proteins that are not covered by any one of CYC2008 complexes is derived from their direct neighbors. The results, as listed in the middle part of Table 1, are proved to be better than that of DCS, which suggests that the accuracy of function prediction can be improved by extending some proteins' domain context from their direct neighbors to the known complexes where they belong to.

Moreover, we have also used predicted protein complexes generated by various clustering algorithms including the widely used IPCA [27], MCODE [28], CPM [29], DPClus [30] and HC-PIN [39] for the usage in DSCP. Here we list the best results of DPClus, in which 62.45% of the 1808 proteins annotated by GO terms in MF, 60.86% of the 3868 proteins annotated by GO terms in CC and 61.27% of the 3909 proteins annotated by GO terms in BP are included in the 965 complexes. The results are shown in the bottom par of Table 1.

As can be seen, the performances of DSCP decrease contrarily when using predicted protein complexes even though the protein coverage increases. This is easy to understand since clustering algorithms cannot predict accurate protein complexes because of many false positive and false negative complex members. Consequently we use the CYC2008 data for the algorithm DSCP afterwards.

**Figure 4:**
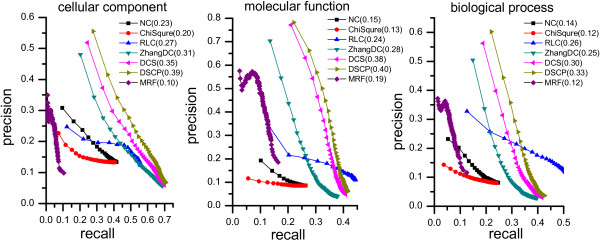
**The precision-recall curves of DCS and DSCP compared to other five existing algorithms.** The figure presents the precision-recall (*PR*) curves of DCS, DSCP and other five existing algorithms (NC, Chi-square, Zhang-DC, MRF and RLC) based on the average prediction performance over all testing protein. The horizontal and vertical coordination of the precision-recall curves are the values of recall (denoted as *TPR*) and precision (denoted as *PPV*). Here, the result of DSCP is obtained by using CYC2008 protein complexes which are determined experimentally. The digits in legend are the maximum *F*-measures for all methods on corresponding GO annotation category (CC, MF and BP).

Furthermore, we perform a comparison of our algorithms of DCS and DSCP with algorithm Zhang-DC [24] which has also used domain context similarity, the other two classical neighbor counting [10] (denoted as NC) and Chi-square algorithms [11], MRF [7] which integrates protein complex, domain and PPI for protein function prediction, and RLC [16] which is a recently proposed method and possesses good prediction performance. To depict the comparison results, precision-recall (*PR*) curve is made used of, whose horizontal and vertical coordination are the values of *TPR* and *PPV*, respectively. The leave-one-out cross validation is carried out on all these methods to evaluate their effectiveness. Our methods DCS and DSCP as well as Zhang-DC infer functions of an unknown protein from its top *K* similar known proteins ranked by these methods. The functions of these known proteins are regarded as the predicted functions of the unknown protein. Moreover, the similarity scores between the unknown protein and the known proteins should be larger than zero. For NC, Chi-square and RLC, we select top *K* GO terms ranked by these methods as predicted functions of an unknown protein and also ensure that the corresponding scores are larger than zero (protein-GO term relationship score for RLC, GO term frequency score for NC and Chi-Square). Here the parameter *K* ranges from 1 to 50. MRF assigns a function to unknown protein if the probability that the unknown protein has the given function is above a threshold. The threshold for MRF ranges from 1 to 0 decreased by 0.01. For a given testing protein and each threshold or parameter *K*, the *TPR* and *PPV* values can be calculated according to the definition in Equations (9) and (10). The final *PR* curves of each comparing method are plotted based on the average *TPR* and *PPV* values over all testing proteins [40]. Figure 4 shows that the *PR* curves of all methods and the digits in legend are the maximum *F*-measures for all methods on corresponding GO annotation category (CC, MF and BP).

As shown in Figure 4, DSCP using CYC2008 protein complex data and DCS achieve the first and the second maximum *F-measures* among all comparing methods on each GO annotation category. For the GO terms in category CC, the *PR* curves of DSCP and DCS are above that of all comparing methods, which means that our methods have a higher number of true positives and at the same time a smaller number of false positives when selecting different thresholds. For the GO term in categories MF and BP, the *PR* curves of DSCP and DCS are above that of the other four existing methods (NC, ChiSquere, ZhangDC and MRF) and are also above that of RLC when inferring functions form a small number of the most similar proteins. However, the precision values of DSCP and DCS drop sharply when inferring function from a large number of similar proteins. Since a lot of functions of these similar proteins will be assigned to the unknown proteins, which will introduce many false positives. The precision values of RLC drop slowly with decrease of the threshold of protein-GO term relationship score. Because decreasing of the threshold slightly increase the number of predicted functions for an unknown protein.

### Prediction on different datasets

There are many databases of PPI network data available online and different datasets vary from each other a lot. Therefore we have tested our new algorithm DSCP on four different scale networks that are obtained from different sources of online databases.

To reduce the influence of noise data, we also remove the duplicated interactions, self interactions and interactions associated with proteins that are functionally unknown or cannot be mapped to UniProtKB/Swiss-Prot entries. As a result, the four datasets are MIPS [41] PPI which contains 4,546 proteins and 12,319 interactions, DIP PPI used previously in this paper that is consisted of 5,088 proteins and 22,277 interactions, BioGrid [42] physical PPI (denoted by BioGrid-phy) which includes 5,759 proteins an 63,084 interactions and the complete network from BioGrid comprising both the physical interactions and the genetic interactions (denoted by BioGrid-cpl), which increases up to 5,985 proteins and 183,228 interactions. The protein complex data used here in DSCP on all the datasets are the CYC2008 protein complexes.

Table 2 shows the results of DSCP, DCS and Zhang-DC when they infer functions from the most similar one known protein (*K* = 1). RLC and MRF show the results when they achieve the maximum F-measure based on average prediction performance on each testing GO term. The results in Table 2 prove that DSCP can always produce the best results followed by DCS on the four datasets in terms of *PPV*, *F*-measures and *MCC* values. The difference between DCS and Zhang-DC rises from whether or not the domain context similarity includes the similarity of proteins themselves. The improvement of DCS proves the effectiveness of our strategy. Both DSCP and MRF utilize protein complex and protein domain information to predict functions for proteins. However the *F*-measure values of DSCP are obviously higher than that of MRF. For example, on DIP network, the *F*-measure values of DSCP are 0.16, 0.3 and 0.23 higher than that of MRF for MF, CC and BP category respectively. It is caused by that MRF infers functions for a protein from its direct neighbors and highly depends on the completeness of domain and complex information from known proteins. By compared with RLC, its performance is inferior to DSCP and DCS, and comparable to Zhang-DC. Since RLC annotates function for unknown proteins by integrating GO term similarity and global PPI network information, it can cover more true functions and has relative higher *TPR* values. However the proportion of positive results are relative lower, which leads to its lower *PPV*, *F*-measure and *MCC* values. All of facts prove that our methods propose an effective strategy for combining protein domain information, protein complexes information and PPI network and outperform other existing methods in function prediction.

**Table 2:** The prediction results based on different protein-protein interaction data

**Data set**	**Methods**	**MF**					**CC**					**BP**				
		**#GO**	**PPV**	**TPR**	**Fmeasure**	**MCC**	**#GO**	**PPV**	**TPR**	**Fmeasure**	**MCC**	**#GO**	**PPV**	**TPR**	**Fmeasure**	**MCC**
MIPS	DSCP	83	0.49	0.41	0.44	0.44	113	0.43	0.40	0.41	0.40	249	0.37	0.37	0.37	0.36
	DCS	83	0.42	0.34	0.38	0.37	113	0.30	0.28	0.29	0.28	249	0.30	0.31	0.30	0.30
	Zhang-DC	83	0.24	0.19	0.21	0.20	113	0.21	0.21	0.21	0.20	249	0.23	0.24	0.24	0.23
	MRF	83	0.36	0.22	0.27	0.28	113	0.19	0.11	0.14	0.14	249	0.18	0.13	0.15	0.15
	RLC	83	0.19	0.32	0.24	0.23	113	0.20	0.37	0.26	0.25	249	0.16	0.37	0.22	0.22
DIP	DSCP	95	0.47	0.40	0.43	0.43	124	0.45	0.43	0.44	0.43	267	0.38	0.37	0.38	0.37
	DCS	95	0.45	0.38	0.41	0.41	124	0.37	0.35	0.36	0.35	267	0.34	0.34	0.34	0.33
	Zhang-DC	95	0.28	0.25	0.26	0.26	124	0.28	0.31	0.29	0.28	267	0.25	0.27	0.26	0.25
	MRF	95	0.33	0.24	0.27	0.27	124	0.19	0.12	0.14	0.14	267	0.20	0.13	0.15	0.15
	RLC	95	0.18	0.34	0.24	0.22	124	0.19	0.46	0.27	0.27	267	0.14	0.41	0.21	0.22
Biogrid-phy	DSCP	103	0.48	0.42	0.45	0.44	130	0.46	0.46	0.46	0.45	299	0.40	0.39	0.40	0.39
	DCS	103	0.45	0.41	0.43	0.42	130	0.40	0.44	0.42	0.41	299	0.36	0.37	0.37	0.36
	Zhang-DC	103	0.32	0.29	0.31	0.30	130	0.35	0.42	0.38	0.37	299	0.30	0.32	0.31	0.30
	MRF	103	0.48	0.26	0.34	0.35	130	0.21	0.14	0.17	0.16	299	0.38	0.13	0.19	0.22
	RLC	103	0.15	0.29	0.20	0.17	130	0.16	0.50	0.25	0.25	299	0.25	0.34	0.29	0.25
Biogrid-cpl	DSCP	105	0.48	0.42	0.44	0.43	130	0.45	0.46	0.45	0.45	303	0.40	0.40	0.40	0.39
	DCS	105	0.43	0.40	0.41	0.41	130	0.39	0.42	0.41	0.40	303	0.36	0.39	0.37	0.36
	Zhang-DC	105	0.30	0.26	0.28	0.27	130	0.32	0.38	0.35	0.34	303	0.26	0.31	0.28	0.27
	MRF	105	0.74	0.25	0.38	0.43	130	0.52	0.13	0.21	0.26	303	0.45	0.13	0.20	0.24
	RLC	105	0.32	0.26	0.29	0.23	130	0.35	0.35	0.35	0.29	303	0.22	0.35	0.27	0.25

This table shows prediction results of five algorithms (DCS, DSCP, Zhang-DC, MRF and RLC) based on four different protein-protein interaction data (MIPS PPI, DIP PPI, BioGrid physical PPI and BioGrid PPI comprising both physical and genetic interactions), including the number of GO terms of each category (MF, CC and BP) on each interaction data set and the corresponding values of *PPV*, *TPR*, *F*-Measure and *MCC*.

### Leave-percent-out cross validation

In the former sections, we used leave-one-out cross validation to demonstrate the algorithms’ improvements made on predicting protein functions. However, in practical applications, there are usually much more proteins without annotations rather than solely one unknown protein. As a result, we next used the leave-percent-out cross validation, which is also a widely accepted validation method [33,34], to demonstrate the effectiveness of our algorithms on networks with less function information.

We run our predicting programs of DCS and DSCP, Zhang-DC [24] and RLC [16] on the network of DIP PPI 1000 times to get the average values of *PPV*, *TPR, F*-Measure and *MCC*. The percentage is set as 10%, 20%, 50% and 80% respectively and the prediction results are displayed in Table 3. As can be seen in Table 3, our methods remain to generate a relatively high *F*-Measure and *MCC* value when the percent of unknown proteins rises up to 50%. Therefore, DSCP seems to be a suitable method for annotating unknown proteins.

**Table 3:** The prediction results with different percentages of protein annotations removed

**Percent**	**Methods**	**MF**				**CC**				**BP**			
		**PPV**	**TPR**	**F-Measure**	**MCC**	**PPV**	**TPR**	**F-Measure**	**MCC**	**PPV**	**TPR**	**F-Measure**	**MCC**
10%	DSCP	0.75	0.43	0.54	0.41	0.69	0.36	0.47	0.35	0.61	0.37	0.46	0.35
	DCS	0.78	0.40	0.53	0.39	0.64	0.31	0.42	0.30	0.55	0.30	0.39	0.29
	Zhang-DC	0.70	0.25	0.37	0.24	0.61	0.24	0.34	0.23	0.50	0.23	0.32	0.22
	MRF	0.47	0.19	0.27	0.15	0.23	0.20	0.21	0.11	0.25	0.20	0.22	0.13
	RLC	0.29	0.38	0.33	0.23	0.29	0.40	0.33	0.24	0.24	0.33	0.28	0.22
20%	DSCP	0.76	0.42	0.54	0.40	0.69	0.35	0.47	0.34	0.60	0.36	0.45	0.34
	DCS	0.75	0.41	0.53	0.39	0.64	0.30	0.40	0.28	0.55	0.30	0.39	0.29
	Zhang-DC	0.71	0.25	0.36	0.24	0.62	0.25	0.36	0.24	0.52	0.24	0.32	0.22
	MRF	0.37	0.21	0.27	0.13	0.24	0.19	0.21	0.10	0.26	0.21	0.23	0.13
	RLC	0.31	0.36	0.33	0.22	0.32	0.38	0.34	0.24	0.25	0.32	0.28	0.22
50%	DSCP	0.78	0.35	0.49	0.34	0.70	0.31	0.43	0.30	0.60	0.31	0.41	0.30
	DCS	0.77	0.33	0.46	0.32	0.66	0.25	0.37	0.24	0.56	0.26	0.36	0.25
	Zhang-DC	0.74	0.20	0.31	0.19	0.65	0.22	0.33	0.21	0.54	0.21	0.30	0.20
	MRF	0.35	0.18	0.24	0.11	0.22	0.16	0.18	0.08	0.22	0.17	0.19	0.09
	RLC	0.40	0.29	0.33	0.19	0.40	0.31	0.35	0.21	0.33	0.26	0.29	0.18
80%	DSCP	0.84	0.24	0.37	0.23	0.76	0.23	0.36	0.23	0.66	0.23	0.34	0.22
	DCS	0.83	0.22	0.35	0.21	0.72	0.18	0.29	0.17	0.63	0.18	0.28	0.17
	Zhang-DC	0.83	0.12	0.21	0.11	0.72	0.16	0.27	0.16	0.63	0.15	0.24	0.14
	MRF	0.32	0.09	0.14	0.06	0.18	0.08	0.11	0.04	0.16	0.09	0.11	0.04
	RLC	0.59	0.17	0.26	0.12	0.58	0.20	0.29	0.14	0.52	0.16	0.25	0.12

This table shows prediction results of four algorithms (DCS, DSCP, Zhang-DC and RLC) by using leave-percent-out cross validation. The percentage is set as 10%, 20%, 50% and 80%, respectively.

## Conclusions and discussion

In this paper, we have proposed an algorithm DCS for protein function prediction by using domain combination similarity to estimate the function similarities between proteins. In addition, we have used the protein complexes to expand domain context scope, and consequently made a desirable improvement to the final prediction results. Our experiments have demonstrated that the Pfam domain data is a useful resource for protein function prediction and its advantages become increasingly obvious as the data grows more complete. In addition, we have illustrated our new algorithm DSCP to be robust when a large percentage of the proteins in the network are unknown. In a word, the algorithm of DSCP described in this paper can be an effective approach for predicting functions for unknown proteins. Additionally, the functional similarity of two proteins measured by our methods DCS and DSCP can be easily used to weight PPI network and plugged into NC, Chi-square, RLC and MRF. In the future work, we will focus on designing more effective method based on the weighted PPI network to predict protein functions.

Like many other similarity-based algorithms, when there is more than one known protein with the same similarity value to an unknown protein, we have met difficulties in choosing a proper reference. How to a construct a good similarity definition with the minimum conflicts remains an important problem to be solved in the future. Besides, in our algorithms the unknown proteins always refer to the same most similar protein whichever GO term is being considered. Mining out the differences between two proteins with a large similarity by using other biological information, such gene expression profiles [43,44] can be a way to get more accurate predictions.

Since proteins can function variously in different organisms or organs, the protein function prediction becomes much more complex when the surrounding environment changes [45]. Moreover, there exist noises in the high-throughput data [46] and are lack of an appropriate evaluating benchmark for different algorithms, which makes the prediction task even more complex and difficult. Consequently, there are many obstacles to overcome and much knowledge remained to be discovered in the field of protein function prediction.

## Competing interests

The authors declare that they have no competing interests.

## Authors' contributions

JC obtained the protein-protein interaction data, GO annotation data and domain data. JXW, JC, WP, ML and LC designed the new methods and analyzed the results. JXW, JC and WP drafted the manuscript together. FXW participated in revising the draft. All authors have read and approved the manuscript
